# The Impact of Polycrisis on Healthcare Systems—Analyzing Challenges and the Role of Social Epidemiology

**DOI:** 10.3390/healthcare13161998

**Published:** 2025-08-14

**Authors:** Agata Wypych-Ślusarska, Karolina Krupa-Kotara, Jerzy Słowinski, Antoniya Yanakieva, Mateusz Grajek

**Affiliations:** 1 Department of Epidemiology, Faculty of Public Health in Bytom, Medical University of Silesia in Katowice, 40-055 Katowice, Poland; kkrupa@sum.edu.pl (K.K.-K.); jslowinski@sum.edu.pl (J.S.); 2 Health Promotion Faculty, Cracow Higher School of Health Promotion Having Its Registered Office in Cracow, 31-158 Cracow, Poland; 3 Faculty of Public Health, Research Institute of Innovative Medical Science, Medical University–Sofia, 1431 Sofia, Bulgaria; a.yanakieva@foz.mu-sofia.bg; 4 Department of Public Health, Faculty of Public Health in Bytom, Medical University of Silesia in Katowice, 41-902 Bytom, Poland; mgrajek@sum.edu.pl

**Keywords:** polycrisis, healthcare systems, social epidemiology, health inequalities, pandemics, armed conflict, climate change, economic crisis, health system resilience, public health policy

## Abstract

In response to contemporary challenges such as the COVID-19 pandemic, climate change, armed conflicts, and economic instability, healthcare systems worldwide are increasingly confronted with multifaceted and overlapping crises—collectively referred to as polycrisis. These interconnected threats amplify one another, placing unprecedented strain on healthcare infrastructure, governance, and equity. The COVID-19 pandemic alone led to an estimated 16.3 million missed hospitalizations in 2020 and 14.7 million in 2021, revealing systemic vulnerabilities and deepening social inequalities. Armed conflicts, such as in Syria and Gaza, have devastated healthcare access. In Gaza, by mid-2024, 85% of the population had been forcibly displaced, with only 17 of 36 hospitals partially functioning and over 885 healthcare workers killed. Climate change further exacerbates health burdens, with over 86% of urban residents globally exposed to harmful air pollution, contributing to 1.8 million deaths annually. This study introduces a novel perspective by applying social epidemiology to the analysis of polycrisis. While the existing literature often emphasizes political or economic dimensions, our approach highlights how overlapping crises affect population health, social vulnerability, and systemic resilience. By integrating sociodemographic and environmental data, social epidemiology supports crisis-resilient care models, targeted interventions, and equitable health policies. We argue for a stronger mandate to invest in data infrastructure, enhance surveillance, and embed social determinants into health system responses.

## 1. Introduction

A polycrisis is characterized by the interaction of multiple crises, which collectively amplify the effects of individual events, thereby creating a complex and challenging problem to manage [1]. The term “crisis” typically denotes a situation where sudden or sequential events pose a significant threat to the welfare and well-being of a large population within a relatively short timeframe, necessitating effective management to mitigate its impact. However, the notion of a polycrisis extends beyond the mere simultaneous occurrence of several crises. The critical aspect lies in the overlapping issues generated by these crises and their mutual interactions within intricate global systems. While individual crises may not always be directly linked, their effects can interact, triggering an interdependent cascade of events. Such interactions can lead to an escalation of problems, where one crisis exacerbates another. Consequently, crises within a polycrisis become synchronized, amplifying negative effects and complicating management efforts. A seemingly minor crisis in one region may thus evolve into a global polycrisis [1].

**Figure 1:**
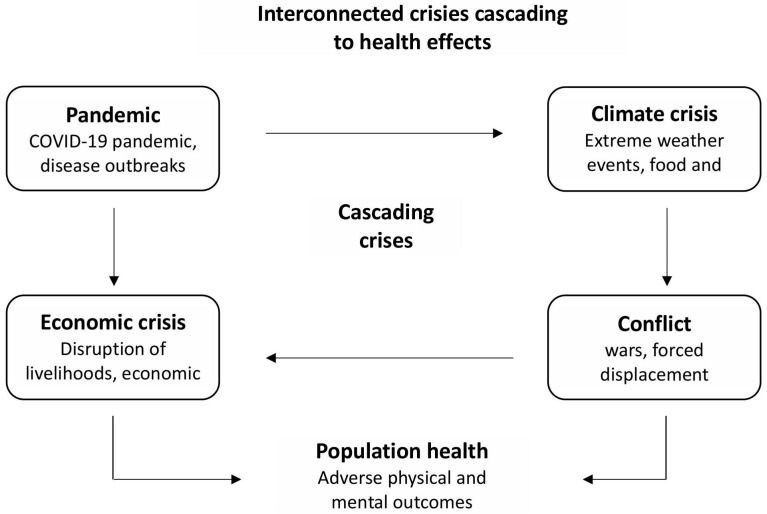
Interconnected crises cascading to health effects.

Contemporary global crises—the COVID-19 pandemic, climate change, economic downturns, and armed conflicts—do not operate in isolation (Figure 1). They create a system of interconnected vessels in which each crisis can initiate or exacerbate others, leading to a cascading effect with serious consequences for public health. For example, the COVID-19 pandemic has led to global economic disruptions, increased social inequality, and strained healthcare systems. Rising unemployment and social isolation have intensified mental health issues and mobility restrictions have hindered access to medical care. The necessity to focus on combating the SARS-CoV-2 virus has disrupted and slowed the achievement of the Sustainable Development Goals (SDGs), particularly in areas related to environmental protection, poverty alleviation, and equal access to healthcare. Economic crashes, often precipitated by pandemics or climate disasters, consequently lead to reduced financing for healthcare systems, increased poverty, and deteriorating living conditions. This, in turn, increases the population’s vulnerability to diseases and stress. Wars and conflicts destabilize regions, destroy health infrastructure, cause migration, and increase the risk of epidemics. Conflicts are often linked to battles over resources, the availability of which worsens owing to climate change and economic crises.

The mutual interaction of these crises leads to a synergistic deterioration in public health. Populations exposed to multiple stressors, for instance, people living in regions affected by war, drought, and pandemics, show higher rates of chronic diseases, mental disorders, and mortality.

A polycrisis may encompass economic, social, political, or climate crises, as well as health emergencies arising from epidemics and pandemics, and increasingly frequent armed conflicts [2].

In the 21st century, the world has encountered individual crises that frequently span multiple regions or significant portions of the globe. The financial crisis of 2008, originating in the United States, rapidly escalated into a global economic crisis. Similarly, the eruption of the Eyjafjallajökull volcano in Iceland in 2010 caused extensive disruptions in air travel across Europe, impacting millions of passengers and global supply chains. The Ebola epidemic and the subsequent COVID-19 pandemic have further exposed the intricate challenges faced by various healthcare systems [1]. The escalation of armed conflicts, including Russia’s invasion of Ukraine and the situation in the Gaza Strip, has disrupted food supplies, precipitating humanitarian and food crises, threatening famine, and increasing the risk of normalizing attacks on healthcare facilities and personnel.

Currently, the discourse on co-occurring crises increasingly revolves around the concept of a polycrisis, with considerations extending to the health impacts on populations. Historically, the notion of a polycrisis has been primarily examined from an economic perspective. However, the prevailing geopolitical climate suggests that the ongoing polycrisis has profound implications for global public health, exacerbating health inequalities that will significantly affect future generations. In light of contemporary challenges such as the COVID-19 pandemic, climate change, and economic and social crises, healthcare systems worldwide must address multifaceted crises, collectively termed a polycrisis [1]. The potential complications arising from these crises, including conflicts and the collapse of public infrastructure and services, present unprecedented challenges with extensive consequences. Consequently, it is imperative to implement measures aimed at fortifying the global preparedness system for such risks. Although numerous countries have developed new capabilities and skills, the current level of preparedness to effectively respond to impending threats remains inadequate.

In the face of escalating polycrises, it has become essential to adopt an interdisciplinary approach when analyzing their impact on population health. One of the key fields enabling such an approach is social epidemiology, which examines the relationships between social factors and the health status of individuals and social groups. Social epidemiology is the branch of epidemiology that focuses on the social determinants of health. It studies how social structures, relationships, and conditions—such as income, education, race, gender, employment, housing, and social support—affect the distribution of health and disease across populations. Rather than examining health outcomes in isolation, social epidemiology seeks to understand the broader societal mechanisms that produce and reinforce health inequalities [3]. Unlike classical epidemiology, which focuses on the distribution of diseases and biological factors, social epidemiology considers the structural determinants of health, such as social inequalities, living conditions, access to healthcare, education, and the influence of public policy. In the context of polycrises, social epidemiology plays both an analytical and prognostic role, enabling the identification of groups that are particularly vulnerable to the cumulative effects of crises. Social epidemiology provides tools for analyzing these complex relationships, making it possible to design evidence-based health interventions, monitor the effects of public policies, and support the equitable allocation of resources. Integrating knowledge from sociology, psychology, economics, and public health allows for a comprehensive understanding of health as a result of the interaction between the individual and their social environment. In the era of global polycrises, social epidemiology has become both analytical and strategic, serving as a foundation for building social and systemic resilience in the face of complex health threats [4].

This paper aims to examine the impact of complex and overlapping crises—pandemics, armed conflicts, climate change, and economic crises—on the functioning of healthcare systems. In particular, it attempts to assess how these phenomena affect the health of the population and the ability of health systems to respond to cumulative challenges. A key element of the discussion is the role of social epidemiology as an analytical and strategic tool in identifying the mechanisms through which polycrises influence public health.

## 2. Health Crisis Resulting from Pandemic Risk

Pandemic crises undeniably exert significant impacts on public health. Over the past two decades, there has been a notable increase in the incidence of infectious diseases with pandemic potential [1]. Since 2007, the World Health Organization (WHO) has declared several international public health emergencies, including those related to H1N1 influenza, Ebola, polio, Zika, and COVID-19 [1]. Analyses of incidence, hospitalization, and mortality for these diseases reveal substantial health inequalities, potentially stemming from adverse economic conditions and restricted access to healthcare.

An analysis of data concerning the H1N1 influenza pandemic indicates that mortality rates were three times higher in the most deprived 20% of districts in England compared to the 20% least deprived (RR = 3.1, 95% CI 2.2–4.4) [5]. Similar patterns were observed in Canada, where hospitalization during the 2009 H1N1 pandemic was associated with various major social determinants of health. The study demonstrated that high social deprivation (OR = 1.66; 95% CI: 0.79–3.46) and material deprivation (OR = 3.46; 95% CI: 1.65, 7.26) increased the risk of hospitalization [6]. In the United States, individuals experiencing financial barriers to healthcare access reported flu-like symptoms more frequently (OR = 1.63, 95% CI: 1.45–1.82). Furthermore, among patients at high risk of influenza complications, self-reporting of flu symptoms was more prevalent, yet the receipt of antiviral treatment was not more frequent, despite recommendations for its use in this population [6].

Research on the Ebola epidemics (2015–2016 and 2018–2020) and the Zika virus in 2016 also highlighted socio-economic inequalities in incidence and mortality rates. Ebola cases predominantly affected individuals of lower socio-economic status [7]. The authors of the study suggested that this epidemic could have been more effectively prevented or controlled if health interventions had been targeted to areas of extreme poverty and if resources had been allocated to development projects addressing basic needs.

Analyses of studies examining the socio-economic determinants of infectious disease incidence, and consequently health inequalities, consistently arrive at a common conclusion: socio-economic poverty can result in poorer health outcomes through various mechanisms, including limited access to healthcare or infrequent utilization of it [1,8].

The COVID-19 pandemic served as a tangible experience for most of the global population, revealing not only regional but also global health disparities. It underscored the necessity of establishing robust and stable healthcare systems to mitigate the impact of future similar events. In numerous regions worldwide, including highly developed nations, unresolved issues within healthcare and public health were exacerbated by COVID-19, exposing the vulnerabilities of existing systems [8,9,10]. The COVID-19 pandemic significantly intensified existing social inequalities and unveiled serious dysfunctions within the healthcare system [10]. The unexpected surge in healthcare demand due to SARS-CoV-2 infections compelled healthcare systems in many countries to reallocate specific services, implement extraordinary public health measures, and postpone planned hospitalizations and surgical procedures. It is estimated that in 2020, 16,300,000 hospitalizations (95% CI: 14,700,000–17,900,000) were missed, with an additional 14,700,000 hospitalizations (95% CI: 13,100,000–16,400,000) in 2021 [10]. The inadequate supply of personal protective equipment, such as masks and ventilators, reflected insufficient planning and a lack of coordination at both national and international levels. Consequently, the most vulnerable social groups, particularly those of low socio-economic status, were disproportionately and severely affected. Data indicate that individuals with lower incomes and racial groups, such as residents of Black and Indigenous communities, experienced higher infection rates and poorer health outcomes [9]. Furthermore, the pandemic highlighted the issue of neglected care for the elderly in long-term care homes, which became epicenters of the epidemic, where conditions were often unacceptable. These challenges demonstrate that the healthcare system was inadequately prepared for crises and lacked effective policies and actions targeted toward those most in need, thereby exacerbating pre-existing inequalities and disparities in healthcare accessibility and quality [9].

The pandemic has caused serious disruptions in the most stable and robust healthcare systems worldwide. In low- and middle-income countries (LMIC), there was a visible cascade of new health problems overlapping with endemic diseases and other health challenges such as HIV, tuberculosis, malaria, and malnutrition [11,12,13]. Given the weak healthcare infrastructure and growing pressure on healthcare systems resulting from new challenges, the scale and effects of the pandemic in LMICs should be considered in the context of the macro-social determinants of health. In resource-limited countries, the pandemic has led to severe interruptions in the delivery of medical services and exposed numerous deficiencies and gaps in healthcare systems, revealing their inability in times of crisis.

Both the prevention and treatment of infectious and noninfectious diseases experienced major disruptions, which may have been associated with an indirect risk of morbidity and mortality from these diseases [13,14,15]. Many services were delayed, and patients often skipped follow-up visits and urgent interventions because of fear and anxiety caused by the waves of the pandemic. As a result, not only was the continuity of care disrupted but the risk of deterioration in patients’ health also increased. Additionally, rising morbidity rates among healthcare workers have further deepened staffing shortages. Shortages in medicines, diagnostics, and technology have been indicated as major causes of healthcare system disruptions in many countries [13,14,15].

Adaptation to the new pandemic situation also includes oncology patients and oncological care [15]. The relocation of human resources, increased use of telehealth, remote patient monitoring technologies, and changes in treatment and patient care schedules are some of the forced adaptive measures [15]. In the field of cancer prevention as well, many screening services were suspended or significantly reduced [16]. These changes may have caused psychosocial effects in the patients, such as anxiety, stress, and a sense of loss of control. Additionally, patients experience financial difficulties and an increased risk of treatment interruption and withdrawal from further care [15].

The European Observatory on Health Systems and Policies, WHO Regional Office for Europe (WHO/Europe), and European Commission created the COVID-19 Health System Response Monitor (HSRM) to present and analyze effective strategies implemented by various countries, mainly from the WHO European Region, in response to the COVID-19 pandemic. The main focus was on health system responses, but the HSRM also included broader public health initiatives aimed at preventing the transmission of the virus as well as measures in other sectors, such as border control, mobility, and the economy [17].

HSRM analysis has provided an impetus to identify the challenges facing healthcare systems in the face of crises similar to those caused by COVID-19 [17]. The most important factors include the following:

1. Investing in public health: the pandemic revealed a lack of investment in public health in many countries, manifested by an inability to slow the transmission of the virus, track and isolate cases, or by the sharp increase in mortality in nursing homes [18]. The low priority given to public health resulted in poorly or superficially developed pandemic response plans that proved ineffective in the face of a real threat. This situation has affected Spain, Italy, and Greece [19];
2. Workforce support, as the pandemic revealed deficiencies in human resources, which were exacerbated during the fight against COVID-19 by the need to maintain basic healthcare services, as well as by frequent SARS-CoV-2 infections among healthcare workers in the early stages of the pandemic [20];
3. Maintaining financial stability—The pandemic has seriously affected the economies and financial stability of many countries, thereby weakening the financial stability of healthcare systems.
4. Strengthening the management of trust: The pandemic activated many groups based on conspiracy theories and undermined the authority of experts. Strengthening public trust in decision-making bodies is a condition for effective management. However, this aspect has been overlooked in many countries. HSRM analysis showed that in Denmark, Switzerland, and Italy, an increase in trust towards officials and healthcare workers was associated with greater acceptance of measures taken to control COVID-19 [21,22]. These countries reported higher vaccination rates and lower hospitalization and mortality rates.

The pandemic revealed yet another important issue concerning healthcare, namely, it highlighted the significance of primary healthcare in establishing effective and stable systems capable of withstanding future crises [23]. This means that healthcare systems should be able to prepare for crises, effectively absorb them, adapt to changing circumstances, learn from experiences, transform, and emerge from difficult situations. This is aimed at minimizing the negative impacts on public health and ensuring the continuity of medical service delivery.

Primary healthcare is one of the fundamental elements ensuring health equity and includes components such as health education, proper nutrition, access to clean water and sanitation, maternal and child healthcare, immunization, treatment of common diseases, and provision of essential medicines. Its aim is to provide protective, preventive, therapeutic, and rehabilitative services that are as close to the community as possible [23]. These goals and actions have been disrupted by the COVID-19 pandemic. Therefore, a key challenge is building resilient primary healthcare (PHC) systems that can anticipate, prevent, adapt, and transform in the face of crises, thus ensuring the continuity of routine health services [24].

The effectiveness of pandemic responses has varied significantly depending on the capacity of national health systems and the political structures in place. Countries with strong public health infrastructure and coordinated governance—such as Denmark and Switzerland—demonstrated greater resilience, higher public trust, and better health outcomes, including higher vaccination rates and lower mortality [24]. In contrast, nations with underfunded public health sectors and fragmented political decision-making, such as Spain, Italy, and Greece, faced greater challenges in slowing transmission and managing healthcare demand [24].

Even countries that scored well on traditional health security and universal health coverage indicators struggled to manage the risks posed by COVID-19. The World Health Organization emphasized that the burden of these challenges was disproportionately borne by vulnerable communities, regardless of national income level [25]. This highlights the importance of systemic preparedness, investment in public health, and transparent governance in shaping effective responses to health emergencies.

## 3. Political Crisis—Armed Conflicts

Peace is crucial for ensuring a healthy and productive community at the global level. Two of the Sustainable Development Goals (SDGs), 16 and 3, aim to promote just, peaceful, and inclusive societies as well as to ensure health and well-being for all. The ongoing armed conflicts around the world, along with the emergence of new ones and the escalation of old ones, make it impossible to achieve the SDGs by 2030 given the current state of progress, or lack thereof [26]. The latest report (2025) on SDG implementation indicates that none of the 17 defined goals will be achieved by 2030, and several goals will be particularly delayed, showing little or no progress since 2015 [26]. Among these is Goal 16: “Promote peaceful and inclusive societies for sustainable development, provide access to justice for all and build effective, accountable and inclusive institutions at all levels” [26].

Since 2015, the number of armed conflicts has increased significantly worldwide. In 2023, a record 59 conflicts involving states were recorded [27]. This increase is the result of both new conflicts and escalation of existing conflicts. Some of the most intense conflicts include the war in Ukraine, the Israeli-Palestinian war, the civil war in Sudan, as well as conflicts in Myanmar and Ethiopia [28].

War can be described as a prolonged public health crisis, affecting not only international relations, but also the health and well-being of individual nations. Regarding health status, the direct effects of war, such as fatalities, the number of wounded, and disabilities, seem to be just the tip of the iceberg. Conflicts also contribute to far-reaching and indirect health consequences resulting from a constellation of various risk factors and cultural and social contexts. Indirect health threats may arise from disruptions to society’s functioning, such as destroyed infrastructure and limited access to medical care [29]. Forced population displacement and the need to live in temporary refugee camps have often led to outbreaks of infectious diseases and the emergence of epidemic hotspots. Factors that increase vulnerability to illness include food and water shortages, which ultimately result in malnutrition and a weakened immune system [29]. Armed conflicts can disrupt vaccination programs and other public health initiatives, increasing the risk of preventable disease outbreaks.

War is also associated with serious mental health consequences, including the risk of developing post-traumatic stress disorder (PTSD), depression, anxiety, and other disorders. A lack of access to appropriate psychological and psychiatric care can exacerbate these problems [1].

Armed conflicts can ultimately have a negative impact on the availability and quality of medical services and the ability to access them. A growing issue in areas affected by armed fighting is the increasing number of attacks on medical facilities and healthcare personnel. Direct attacks on hospitals, clinics, and healthcare infrastructure disrupt or completely halt the functioning of these facilities, forcing staff members to leave their workplaces. This limits access to basic healthcare and interrupts the continuity of preventive programs, such as children’s immunizations. Access to basic healthcare is an indicator of the effectiveness of the healthcare system [30]. Meanwhile, an increasing number of countries affected by conflict and the erosion of adherence to international humanitarian treaties are weakening these systems in many parts of the world. A study conducted in Burkina Faso found that conflict, in all its forms and manifestations, significantly affects health services for women and children [31]. It is worth taking a closer look at this conflict and its impact on the healthcare system, especially since Burkina Faso has implemented several effective solutions [32,33]. Unfortunately, these were interrupted by ongoing conflicts, which escalated in 2019. Until then, the country was on track toward universal health insurance, and the reach of health services expanded up to the point of conflict escalation. A particularly important aspect of the reforms was maternal and child healthcare as well as prenatal care [32,33]. The government introduced a series of reforms aimed at improving financial protection and increasing the reach and accessibility of healthcare. In Burkina Faso, fees for healthcare services were abolished for children under 5 years of age, as well as for pregnant and breastfeeding women. In 2019, prior to the escalation of the conflict, 79% of the pregnant women participated in at least four prenatal care visits. Progress was also evident in other indicators; maternal mortality dropped to 283 per 100,000 live births, while under-five mortality reached 87 per 1000 live births [31]. Unfortunately, high-intensity conflict events led to the emergence and intensification of negative effects in the medical services sector: prenatal care visits decreased by 3.9%, facility-based deliveries by 7.2%, cesarean sections by 9.4%, postnatal visits by 4.3%, outpatient visits for children under five by 7.2%, and by 12.0% for children aged 5–14 years [31]. The findings from Burkina Faso highlight the fragility of healthcare systems in unstable countries. Expanding the reach of healthcare is a positive factor that can genuinely improve health indicators; however, other factors or barriers can quickly undermine these positive effects. The growing level of uncertainty, lack of security, and Islamic jihadist insurgency have significantly limited the population’s ability to access healthcare and obtain medical services.

A similar situation can be observed in other regions affected by conflict. A study in Nepal showed that women are less likely to use perinatal care during periods of intense conflict [34]. Likewise, observations from northeastern Nigeria, northern Uganda, and Liberia yielded the same conclusions [35,36,37]. In Mexico, during the Zapatista armed conflict, most births took place at home because of decreased access to emergency obstetric care [38].

The long-standing conflict in Syria has had a devastating impact on the civilian population, infrastructure, and access to healthcare [39]. The healthcare system in northwestern Syria has been severely disrupted. Since 2012, the Damascus Ministry of Health has withdrawn its management from all areas controlled by the opposition, leading to serious shortages of resources, including a lack of medical personnel [40]. Additionally, the system suffers from numerous attacks on medical facilities and the absence of a central body to coordinate the healthcare activities in the region. These attacks have been among the main wartime tactics often used in these areas [41,42,43]. As a result, over 27% of Syria’s population live in areas with a shortage of healthcare workers, which seriously impedes access to medical care [44].

Russia’s invasion of Ukraine has also provided evidence of non-compliance with international agreements. One of the main objectives of the Russian strategy is to destroy the Ukrainian healthcare system, which seriously violates Ukrainians’ right to health [45]. In the first two weeks of the war, four to five hospitals and clinics were affected every day. By the end of 2022, 707 incidents were recorded, including damage to almost 9% of the country’s hospitals as well as attacks on pharmacies, blood donation centers, dental clinics, and research institutions. Sixty-five ambulances and eighty-six medical workers were also affected; 62 people were killed and 52 were wounded, although these numbers may have been underestimated [45]. The hospital in Sievierodonetsk was shelled multiple times, and witnesses reported the use of drones before the attacks, indicating deliberate action. In regions controlled by Russia, doctors were intimidated, held hostages, and forced to cooperate, while some who were imprisoned experienced torture and inhumane treatment [45].

Despite the efforts of Ukrainians, the WHO, and other organizations, the quality of healthcare has deteriorated, and many facilities lack access to running water or electricity. In eastern Ukraine, hospitals can only provide limited care, forcing patients to travel long distances or forgo treatment. Since the war began, the number of routine childhood vaccinations has dropped, increasing the risk of disease outbreaks such as polio, measles, or diphtheria. While access to medications is maintained in areas controlled by Ukraine, there is a lack of information regarding access to HIV therapy and other services in regions occupied by Russia. Treating chronic illnesses has become increasingly difficult, and many displaced people have stopped taking essential medications. This conflict has also had a serious impact on the mental health of both civilians and soldiers, causing widespread trauma [45].

In the face of climate change, war, and conflict, approaches to protecting medical personnel involved in humanitarian missions have changed. A hospital is no longer one of the safest places in the conflict zone. Armed groups deliberately attack medical staff and deny aid to selected population groups. Attacks on healthcare facilities have serious consequences for the functioning of the care systems. Losses in infrastructure and interruptions in electricity and water supply significantly limit the ability to provide acute, preventive, and routine care [46]. For example, over a million suspected cases of cholera have been recorded in Yemen, illustrating how war affects the spread of infectious diseases. The infant mortality rate usually rises by approximately 13% during five-year conflicts. Most wartime deaths are caused by malnutrition and diseases rather than by direct combat [46].

Although healthcare facilities and medical personnel are protected by international law, which prohibits deliberate attacks on medical infrastructure, rights safeguarding healthcare were established in the 1949 Geneva Conventions and their protocols, forming the foundation of international humanitarian law [47]. Their purpose is to ensure that even in times of conflict, people can access medical care without fear of safety. Thus, deliberate attacks on medical services constitute a violation of these rights and, in some cases, may amount to war crimes. All countries have committed to complying with these regulations. In 2016, the UN Security Council condemned attacks on healthcare and called for actions to stop them [48].

Unfortunately, attacks on medical facilities and personnel are still ongoing, posing a serious threat to the functioning of healthcare systems worldwide. Reports from various conflicts often show that hospitals are being bombed and healthcare workers are being attacked [49]. The increased use of explosive weapons in populated areas increases the risk to civilians and infrastructure, including healthcare. Often, these attacks are either intentional or dismissed as “collateral damage,” even if they violate international law. Since the beginning of 2024, the WHO has confirmed nearly 700 attacks on medical facilities and staff in Ukraine and Palestinian territories, resulting in more than 500 injuries and nearly 200 deaths [50]. The situation in Sudan and Myanmar is similar—hospitals and clinics are being targeted, making it impossible for millions of people to access basic healthcare [49].

Such violence can lead to a near-total collapse of the healthcare system. For example, by January 2024, as many as 84% of facilities in the Gaza Strip had been damaged or destroyed, making it impossible to access essential care, worsening chronic illnesses, and promoting the spread of diseases [51].

The destruction of healthcare in the Gaza Strip represents one of the most severe humanitarian disasters resulting from war [52,53]. Since the outbreak of the conflict in October 2023, more than 40,000 people, including hundreds of healthcare workers, have died. By the end of May 2024, almost the entire population of Gaza (approximately 1.93 million people, or 85%) had been forcibly displaced, leading to serious damage to medical infrastructure [53]. Many displaced people are forced to live in temporary, overcrowded, and unsanitary shelters where basic necessities such as food, water, and access to healthcare are lacking. As of September 2024, only 17 out of 36 hospitals were operating to a limited extent, and there were severe shortages in medical supplies. Additionally, 130 ambulances were damaged, and around 885 healthcare workers were killed while on duty [53]. People in Gaza are trapped in a dangerous cycle, in which malnutrition and disease fuel one another. Everyday illnesses can become death sentences, especially in children. Malnutrition weakens the body, making it harder to heal injuries and fight off common infections, such as diarrhea, pneumonia, or measles. In turn, these infections increase the body’s need for nutrients while limiting their absorption, deepening malnutrition. The lack of access to medical care has led to a decrease in vaccinations, and access to clean water and sanitary conditions is extremely limited. Concerns about children’s health are growing, and the risk of severe illness and death, especially among children with severe acute malnutrition who urgently need help, is very high [52,53].

The case of Gaza illustrates the theoretical framework of “structural violence,” demonstrating how systemic inequalities and geopolitical factors inflict suffering on masses deprived of basic resources. Conflict and forced displacement worsen the health of the population, especially in vulnerable groups such as children, women, and people with chronic illnesses. Sanitary conditions in Gaza are extremely poor, fostering outbreaks of infectious diseases such as typhoid fever, which results from a lack of clean water and proper hygiene. These types of threats are not unique to this area; similar crises occur in other conflict-affected regions. Additionally, armed conflicts increase the risk of health crises, and children who experience and witness violence are more likely to suffer from PTSD and depression, further hindering their normal development [54].

While individual case studies provide valuable insights, broader generalizations are essential to understand the systemic impact of armed conflicts on health. According to WHO estimates, infant mortality rates increase by approximately 13% during five-year conflicts, and most wartime deaths result from malnutrition and preventable diseases rather than direct combat [55]. In conflict zones, per capita health spending often drops significantly due to infrastructure destruction and resource diversion. For example, in Syria, health expenditure per capita fell by more than 40% between 2010 and 2016 [55]. These indicators highlight the scale of disruption and underscore the need for resilient health systems capable of maintaining essential services under extreme conditions.

The causal mechanisms linking conflict to health deterioration are multifaceted. Armed violence leads to displacement, infrastructure collapse, and disruption of supply chains, which in turn reduce access to care and increase exposure to health risks. Health system resilience—the ability to absorb, adapt, and transform in response to crises—is crucial in mitigating these effects [56]. Countries with decentralized health governance and strong primary care networks, such as Colombia and Rwanda, have demonstrated greater adaptability during conflict-related disruptions [57]. Strengthening resilience requires investment in workforce protection, mobile health units, and flexible service delivery models that can operate in unstable environments.

Although examples of healthcare system disruptions during political crises and armed conflicts are compelling, the literature often lacks sufficient grounds for generalization. There is a noticeable absence of standardized analytical frameworks and cross-context comparisons, which limits the ability to extrapolate findings beyond individual case studies.

Quantitative data supporting the narrative of health system degradation in conflict settings remain scarce. While some figures on excess mortality and service utilization exist, they are frequently fragmented, context-specific, and lack longitudinal depth. This hinders the development of robust, evidence-based assessments. Moreover, the causal mechanisms through which conflict affects healthcare—such as infrastructure destruction, workforce depletion, and governance breakdown—are typically described in isolation and without comparative analysis. As a result, their relative impact across different geopolitical settings remains unclear. Finally, health system resilience in conflict zones is underexplored. Although some studies reference absorptive, adaptive, and transformative capacities, most analyses are retrospective and descriptive, with limited documentation of long-term strategies or structural reforms.

Considering the situation in areas affected by conflict, several key challenges can be identified that face healthcare systems, international organizations, and public health: lack of access to basic healthcare; limited access to medicines, vaccines, and medical supplies; difficulties in transporting patients and medical personnel; attacks on medical facilities and healthcare workers; obstacles to carrying out medical and health activities; challenges in monitoring and reporting on health situations; difficulties in conducting statistics and epidemiological analyses; delays in responding to disease epidemics and health crises; and overburdened healthcare systems in receiving areas. The situation in war zones significantly hinders the achievement of SDGs related to health and peace and requires international cooperation, rapid emergency response, reconstruction of infrastructure, and support for communities affected by conflict, as well as clear and decisive action against attacks on healthcare facilities and medical personnel to prevent the normalization of such behaviors. Furthermore, addressing the problem of attacks on healthcare systems during conflict is essential for achieving the SDGs.

## 4. Climate Crisis

Climate change is one of the most significant challenges facing the modern world, as well as public health, since it has both direct and indirect impacts on the health of populations and, consequently, on the functioning of healthcare systems [58]. The direct health effects of climate change include deaths caused by extreme weather events such as heat waves and floods. Indirect effects include disruptions in food systems and water availability, which contribute to an increase in zoonotic, waterborne, vector-borne, and mental health problems. Climate change also weakens the ability of healthcare systems to provide universal health coverage. The increasing burden on these systems, related to the need to respond to weather crises and the rise in climate-related diseases, necessitates the modernization of infrastructure, expansion of human resources, and implementation of more environmentally sustainable solutions [58].

Air pollution resulting from the burning of fossil fuels and forest fires increases the risk of respiratory and cardiovascular diseases [59]. Studies indicate that over 86% of urban residents worldwide are exposed to harmful particulate matter, which contributes to approximately 1.8 million deaths annually [60]. Extreme weather events, such as heatwaves, droughts, and floods, cause an increase in cases of heat stroke and fatigue, and exacerbate chronic diseases such as asthma or COPD. For example, during a heatwave in Portugal in 2006, because of a 1 °C temperature increase, COPD incidence rose by 5.4%, especially among older adults and women [61]. Climate change has also affected the spread of new diseases. One example is Mesoamerican nephropathy, a chronic kidney disease that appears in Central America and is linked to repeated dehydration caused by heat stress [62]. These phenomena can lead to the emergence of diseases previously unknown or rarely seen in a given region, posing a challenge for healthcare systems.

Increasingly frequent and intense extreme weather events, such as heatwaves, floods, forest fires, tropical storms, and hurricanes, have contributed to the emergence of humanitarian crises on a global scale. The WHO estimates that currently about 3.6 billion people live in areas that are highly susceptible to the effects of climate change [58]. Forecasts indicate that climate-related impacts can cause an additional 250,000 deaths annually, mainly due to malnutrition, infectious diseases such as malaria and diarrhea, and heat stress [58]. Underdeveloped regions are particularly vulnerable, where healthcare infrastructure is limited, and the lack of adequate resources and support systems increases the risk of widening global health inequalities. Low-income countries and small island developing states (SIDS) have the heaviest health consequences, even though their contribution to global emissions is minimal.

It is increasingly emphasized that the relationship between climate and health is no longer indirect; indeed, it is becoming one of the main factors disrupting the functioning of healthcare systems [63]. The rising frequency and intensity of extreme weather events, such as heat waves, hurricanes, and floods, lead to disruptions in food supply chains and contribute to the increase in zoonotic infections. The evolution of infectious diseases has been directly linked to climate change. According to the Intergovernmental Panel on Climate Change (2022) [64], rising temperatures and milder winters have allowed pathogens to establish themselves in new regions that were previously inhospitable because of climatic conditions, increasing the risk of vector-borne diseases. Moreover, warming seas are causing intensified algal blooms, which can produce neurotoxins and expose marine life, wildlife, and humans to serious health risks. These changes necessitate transformation of traditional healthcare models, focusing on and adapting to the realities of climate medicine [63].

Extreme weather events and climate change disrupt the functioning of the medical infrastructure [52]. Storms, floods, forest fires, and energy crises can destroy or damage healthcare facilities, leading to shortages in supply and interruptions in service delivery. One example is the evacuation of 6300 patients from New York during a superstorm [65]. Disruptions in supply chains, such as the destruction of plants that produce medications or saline solutions, can lead to serious losses in hospitals and put patients’ lives at risk. Additionally, power outages during heatwaves prevent the use of cooling devices and medical equipment, which are particularly dangerous for patients with chronic illnesses requiring ongoing therapy [66]. The risk of patients with chronic diseases is especially serious. Increases in temperature and extreme conditions can worsen the symptoms and increase the risk of death. For example, during heatwaves, patients with heart and respiratory diseases are more prone to complications and their condition can deteriorate rapidly [67]. Furthermore, instability in healthcare systems can lead to delays in cancer detection and treatment. Interruptions in the availability of diagnostic and therapeutic services, such as radiotherapy or screening tests, result in poorer prognosis and lower survival rates [68]. Extreme temperatures can lead to improper storage of medications, particularly in facilities with an insufficient power supply. During heat waves, medications can spoil, reduce their effectiveness, and potentially cause adverse reactions. Research has shown that rising temperatures promote the development of bacterial resistance to antibiotics, which poses a serious challenge for the treatment of infections [69]. Moreover, patients using diuretics or antipsychotic medications, which disrupt thermoregulation, are at risk of overheating and hospitalization during heatwaves [70].

Adapting healthcare systems to climate change also effectively reduces carbon footprints. This requires the implementation of a wide range of ecological actions, such as promoting energy-efficient solutions, reducing waste, and selecting low-emission options. These interventions should be accompanied by systemic transformation strategies, including changes in the delivery and consumption of healthcare services. Sustainable approaches, such as limiting waste generation or promoting low-intensity care, can simultaneously benefit the environment, economy, and public health.

The healthcare sector is a significant source of greenhouse gas emissions, and its role in contributing to climate change may intensify owing to the increasing global demand for healthcare services [71]. Direct emissions result from the operation of healthcare facilities themselves, such as energy consumption, heating, or transport of staff and patients. Indirect emissions originate from energy production, purchasing goods and services, and travel, which require strategies for their reduction. Changes in service delivery organizations, reduced travel, material recycling, choosing less polluting anesthetic gases, and promoting green procurement can effectively reduce emissions. Systemic changes are also necessary to improve the sustainability of healthcare systems. The structure of delivery, level and mode of care organization, technology used in treatment, and coordination of therapy all have a significant impact on carbon dioxide emissions. Optimizing these elements allows for greener and more efficient healthcare delivery, which is the key to achieving both climate and health goals [71].

Climate change poses a serious threat to global health and affects all aspects of social and environmental life. Its consequences exacerbate inequalities and hinder the achievement of development goals, including universal healthcare. Immediate, coordinated action at the international, national, and local levels is necessary to reduce emissions, increase the resilience of health systems, and protect vulnerable communities. The priority is for these institutions to take measures aimed at reducing their own impacts on greenhouse gas emissions, which are the main drivers of climate change [63]. For example, the health sector in the United States generates approximately 8% of carbon dioxide emissions, a significant portion of which is related to particulate emissions and respiratory illnesses caused by particulates. In response, some organizations, such as Kaiser Permanente and the Gundersen Health System, have taken steps toward carbon neutrality, achieving sustainability and energy self-sufficiency status [72]. Similar commitments have been made in the United Kingdom, with a declaration to achieve climate neutrality in the healthcare system by 2040 [73].

While references to extreme weather events and systemic stress are valid, the literature often reiterates these points without deeper analytical development. To address this, we have expanded the section to include existing frameworks and strategic considerations.

The WHO Operational Framework for Building Climate Resilient Health Systems outlines ten key components that enable health systems to anticipate, prevent, and manage climate-related health risks [26]. These include integrated risk assessments, early warning systems, sustainable infrastructure, and workforce capacity building. The framework is particularly relevant for countries developing health components of National Adaptation Plans under the United Nations Framework Convention on Climate Change (UNFCCC).

Despite growing awareness, there is limited discussion in the literature on the trade-offs between mitigation and adaptation strategies. Mitigation efforts—such as reducing healthcare sector emissions—are essential for long-term sustainability, while adaptation measures—such as heatwave response plans or flood-resilient infrastructure—are critical for immediate risk management. Balancing these priorities requires context-specific planning and resource allocation, which is often underrepresented in current analyses.

By incorporating these elements, the revised section aims to provide a more comprehensive and policy-relevant perspective on climate-related health system challenges.

It is necessary to integrate environmental data into clinical practice and public health. Developing effective early warning systems linked to climate events will enable the identification of vulnerable populations and facilitate the implementation of preventive measures. Healthcare systems must adopt a proactive approach to reduce their environmental impact and integrate environmental data into daily clinical and public practice. Such an integrated approach based on scientific evidence and social justice will help mitigate the negative effects of climate change on public health.

## 5. Economic Crisis

Economic crises constitute an important component of the complex landscape of contemporary public health threats [1]. They often coincide with other types of crises (political, climate, and health), amplifying their social and economic impact.

Since the global financial crisis of 2007–2008, many countries have experienced slow economic growth and considerable economic instability. At the beginning of 2008, the European Union began facing one of the most serious debt-related financial crises in its history. Several member states saw a sharp economic downturn, an increase in public sector debt, and difficulties securing funds in international markets. This situation negatively affected the living conditions of citizens who increasingly experienced economic instability resulting from job loss, reduced incomes, and cuts in spending on social security systems [74]. The crisis deepened in 2010 when institutions such as the European Central Bank and the International Monetary Fund were forced to launch aid programs for the most affected countries, including Greece, Portugal, Ireland, and Cyprus. In response to the worsening economic situation, some countries have implemented austerity policies aligned with local conditions and the projected scale of the social consequences. As part of these measures, public spending, including funding for the health sector, was curtailed. Among others, these included pay cuts for healthcare workers, reductions in budgets for medicines and medical services, and changes in staff organization, such as longer working hours. These reforms had significant consequences for the accessibility and quality of medical services, particularly regarding the balance between the system’s efficiency and its social function [74].

Particularly significant consequences for public health have resulted from events in recent years—the COVID-19 pandemic and the war in Ukraine—which have led to a rapid rise in inflation, cost of living, and interest rates, affecting the availability of essential goods such as food and energy [1]. Economic crises that overlap with other types of crises pose threats to public health. Data from numerous European and non-European countries indicate a marked increase in mental health problems among populations affected by the crisis, especially regarding depression, stress, and suicidal behaviors [1,75,76,77,78]. An increased number of self-harm cases and an increase in suicide mortality among socially vulnerable groups have also been observed. For example, in the years immediately following the crisis (2008–2010), a clear increase in the number of suicides was registered in the USA, UK, and Spain [1,78].

The literature emphasizes a strong relationship between job loss and deteriorating mental health, particularly in the context of depression and suicides [1,78,79]. An increase in the unemployment rate at the regional and national levels is directly linked to worsening health indicators and quality of life. Importantly, the negative effects of recession are distributed unevenly, deepening existing health inequalities both geographically and in terms of socioeconomic status [1,78,79].

The crisis and the resulting changes in healthcare systems require special attention, as health policies have a direct impact on the well-being of residents [74]. Numerous studies indicate that during a recession, inequalities in accessing medical care have become more pronounced, especially in the most vulnerable groups, such as the unemployed, women, migrants, seniors, the homeless, people with low education levels, and those with the lowest socio-economic incomes [74,80]. In Greece, older people were particularly affected, including migrants, due to cuts in healthcare funding and deepening inequalities in access to services [81]. In Portugal, the crisis negatively affected the availability of health services and co-payments for medications, limiting the ability to purchase pharmaceuticals, especially among seniors [82].

In European Union countries, the link between the health insurance system and employment (for example, in Greece) meant that a rise in unemployment led to a decrease in the number of insured individuals, which in turn resulted in part of the population being excluded from the healthcare system [74,82]. In other countries, an increase in dual insurance (public and private) was observed. During the crisis, private insurance coverage in households initially increased, compensating for the loss of public benefits, but over time, due to limited financial resources, its level declined, further deepening inequalities in access to healthcare services [74]. Income constraints exacerbated inequalities in access to health services, with the effects being most pronounced in southern and western European countries [74,83]. In Spain, restrictions on access to services mainly affected low-income households, with budget cuts intensifying during the crisis [84]. In Greece, decreased funding for health services led to a reduction in the ability of lower-income households to pay out-of-pocket for healthcare [85].

During periods of economic crisis, many European countries undertook measures aimed at reducing public spending, which significantly affected the functioning of their healthcare systems. One of the most used austerity measures was reducing the number of available hospital beds, which in turn necessitated the closure of wards and restricted access to hospital services [74]. In Spain, the result of these actions was not only a reduction in infrastructural resources, but also longer waiting times for health services, especially in primary healthcare and in regions with varying approaches to health policy [84]. In Ireland, pressure on hospital bed availability led to the need to strengthen the primary healthcare sector and modernize infrastructure [86].

Low- and middle-income countries (LMICs) are disproportionately affected by economic crises, yet they remain underrepresented in many global health analyses. Economic downturns in these regions often exacerbate structural weaknesses, including underfunded health systems and limited fiscal space, which restrict governments’ ability to maintain or expand essential services [87]. Despite the urgency, there is a notable lack of discussion around healthcare financing metrics—such as public health expenditure as a percentage of GDP, out-of-pocket spending, and donor dependency—and opportunity costs, which are crucial for evaluating trade-offs in resource allocation [88].

The financial crisis also had significant consequences for medical personnel. In many countries, restrictions on hiring and wage cuts were introduced, which contributed to a deterioration in working conditions, increased dissatisfaction among employees, and difficulties in accessing specialized medical staff [74,85]. The negative effects of these changes were particularly felt by younger healthcare workers, who often decided to emigrate to countries offering more stable and competitive employment conditions [89].

Additionally, in response to the need to reduce costs, some countries decided to implement systemic reforms including partial privatization of health services and changes in coverage of treatment costs [89]. Although such measures aim to improve system efficiency, in many cases, they have led to reduced service accessibility, especially for people with lower incomes. At the same time, there was an increase in private spending on healthcare, indicating the limited effectiveness of public healthcare during crisis periods [74,90]. In contrast to the general trend of cutbacks, the Netherlands undertook measures to support access to services for groups at risk of health exclusion, a case in point being the expansion of funding for physiotherapy services for people with low incomes [74]. Health system reforms such as privatization are often presented in a binary fashion, typically emphasizing negative outcomes without acknowledging their complexity. Evidence from LMICs shows that privatization can have mixed effects: in some contexts, it has improved efficiency and access to care, particularly in urban areas; in others, it has deepened inequalities and reduced access for vulnerable populations [91].

As a result, the number of people unable to meet their health needs has increased significantly. It is estimated that since the beginning of the COVID-19 pandemic, over 1.5 million people have not received the necessary medical services [74,83]. The main barriers were high service costs (36%) and long waiting times (15%) [86]. The situation was particularly difficult for non-EU migrants, especially the undocumented, who experienced significantly limited access to healthcare in countries such as Spain and Italy [92]. This problem is especially acute among populations with lower socioeconomic status, leading to widening health inequalities. This requires strategic political and social actions aimed at leveling health opportunities and counteracting systemic marginalization in access to healthcare.

Economic crises have a multifaceted impact on public health, particularly through rising unemployment and the deteriorating mental well-being of the population. These phenomena are unevenly distributed and contribute to the intensification of existing health inequalities. In the context of contemporary threats, such as pandemics and armed conflicts, there is an urgent need for systematic monitoring of the economic effects on health and for the implementation of preventive and adaptive measures. In response to the growing risks resulting from overlapping crises, many countries are developing integrated strategies to increase the resilience of their health systems [93]. These measures focus on several key areas. First, investments are being made in infrastructure, including the expansion of medical facilities, laboratories, and information systems, which enables more efficient crisis management and the rapid scaling of services. Simultaneously, epidemiological monitoring and surveillance systems are being developed and designed to detect threats early and coordinate institutional responses. An important component in strengthening systems is the effective management of resources—both materials (e.g., medicines and equipment) and humans. Ensuring adequate stockpiles and flexible deployment of medical personnel allows for the continuity of care in emergency situations. In this context, regular crisis response training for medical staff is essential, as is the implementation of alternative models of service delivery, such as telemedicine, which makes it possible to provide care even when mobility is restricted. In many countries, formal crisis management plans have been developed, covering both national and local levels. Such organizational frameworks enable coherent and swift actions during periods of destabilization.

Resilience of healthcare systems is becoming a key component of public safety. Only strong and flexible health structures can effectively absorb crisis shocks, maintain the continuity of essential medical functions, and limit the social and health impact of such events. Lack of adaptive capacity leads to system overload, reduced access to services, and increased morbidity and mortality. For this reason, building health system resilience should be treated as a strategic priority, not only in the context of current challenges, but also as an investment in the future health stability of societies.

## 6. The Role of Social Epidemiology in the Era of Polycrisis

Social epidemiology is an interdisciplinary field of science that examines the impact of social, economic, and institutional factors on population health [3,4].

The theoretical frameworks of social epidemiology include those that focus on explaining the impact of broadly understood social factors on the health of populations. For example, the Fundamental Cause Theory assumes that socioeconomic status remains a persistent determinant of health, as it is associated with access to resources such as knowledge, money, and social capital, which protect health regardless of time and type of disease [94]. Syndemics Theory emphasizes that co-occurring health problems interact with social and environmental stressors, worsening the health status of marginalized communities [95]. On the other hand, social capital theory points to the importance of social relationships, trust, and community cooperation in shaping the health of individuals and groups [94]. Based on these theories, social epidemiology has developed tools to analyze population health that enable the identification and monitoring of health inequalities. Tools such as the Gini coefficient and Slope Index of Inequality (SII) are used to measure health disparities [96]. Applying these indicators to data from the Global Burden of Disease has revealed persistent relative inequality in disease burden between countries, despite a decline in absolute inequalities. Social network analysis makes it possible to understand how social relationships and structures influence health behaviors, disease transmission, and access to care [97]. This tool has been used in research on HIV prevention, mental health, and management of chronic diseases. Despite the theoretical and methodological richness of social epidemiology, case studies demonstrating its impact on health policy are still limited. However, research initiatives based on collaboration with local communities in the USA have shown that integrating knowledge from social epidemiology can lead to more equitable health interventions [98].

In the context of complex crises, known as polycrises, where health, economics, climate, and social threats overlap and reinforce each other, the role of social epidemiology becomes particularly significant [3,4]. However, under conditions of escalating polycrises, the application of this field faces a range of operational challenges that are crucial for the effectiveness of health interventions.

One of the main problems is the limited access to current and reliable social data during crises, especially in regions affected by armed conflicts, climate disasters, or economic collapse. The lack of consistent systems to monitor the social determinants of health makes it difficult to quickly identify at-risk groups and evaluate intervention outcomes. Additionally, integrating epidemiological data with information from other sectors, such as humanitarian aid, social policy, and crisis management, requires inter-institutional and cross-sectoral collaboration. In crisis conditions, there is often a lack of coordination mechanisms and actions taken are fragmented, failing to consider the social determinants of health.

One solution to these problems could be the development of integrated data platforms that connect epidemiological information with social, economic, and environmental data. Implementing such tools enables a more accurate forecasting of crisis impacts and the design of interventions tailored to local conditions. It is also important to strengthen the competencies of public health teams in social analysis through training and cooperation with experts in sociology, economics, and political sciences. Recognizing social epidemiology as an integral element in public health crisis management is crucial. Its operational application should be embedded in crisis response strategies, both locally and internationally, with particular attention paid to the needs of the most vulnerable populations.

Social epidemiology also serves an analytical and strategic function, making it possible to track health inequalities and direct targeted interventions. It enables the identification of the most vulnerable social groups, such as individuals with low socioeconomic status, undocumented migrants, or residents of regions with underdeveloped healthcare systems. This allows for the precise allocation of resources and targeting of preventive and therapeutic actions, which is key to limiting the deepening of health inequalities during crises.

Social epidemiology also supports the strengthening of healthcare system resilience. Providing data on the weaknesses and strengths of systems in the context of social inequalities facilitates the development of crisis-resistant care models. The analysis of sociodemographic, environmental, and behavioral research enables the integration of knowledge and the creation of flexible healthcare structures such as telemedicine, primary care, and local support resources.

Moreover, social epidemiological tools make it possible to systematically combine data from diverse sources and levels, both individually and institutionally. Harmonizing socioeconomic data with health indicators allows for the ongoing monitoring of the effectiveness of health policies and interventions, supporting the development of resilient systems. Social epidemiology also provides insights into the spread of inequalities and the mechanisms of their emergence, making it possible to assess the outcomes of actions such as the development of primary healthcare, psychosocial support, and the expansion of epidemiological surveillance systems.

Finally, in polycrisis conditions, social epidemiology plays a key role in achieving public health goals. It enables data integration to create health policies aligned with the principle of “health for all.” Thus, it is possible to identify areas that require urgent action, promote effective resource allocation, reduce health inequalities, and realize the goals of sustainable development in health.

Meanwhile, one of the key challenges in analyzing contemporary crises—whether health-related, climatic, economic, or geopolitical—is the lack of systematic consideration of the social epidemiology perspective when assessing their impact on population health. Each of these crises generates unique mechanisms of health inequalities that can be effectively identified and analyzed precisely through social epidemiology tools. Unfortunately, in many scientific studies and strategic documents, the role of this field remains marginalized or treated in a fragmented manner. Including social epidemiology in the analysis of every type of crisis allows not only the identification of the most vulnerable social groups but also the design of interventions tailored to local social, cultural, and economic conditions. For example, during a pandemic, social epidemiology can indicate which communities have limited access to information, testing, or vaccinations. In the context of a climate crisis, it can help us understand how environmental changes affect the mental and physical health of people living in poverty or in threatened areas.

In light of mounting and mutually reinforcing health, climate, economic, and social crises, there is a need to formulate a clear evidence-based mandate for policymakers, health system leaders, and the academic community. This mandate should be based on recommendations from epidemiological research and should recognize the essential role of social epidemiology in responding to complex crisis situations.

Specifically, there is a need to demand:

1. Investment in social health data infrastructure, enabling the integration of information from different sectors and levels, from individual to systemic data, to monitor health inequalities and the effectiveness of interventions.
2. The development of local social and health support systems that are resilient to disruptions caused by crises and tailored to the needs of the most vulnerable social groups, considering cultural, economic, and environmental contexts.
3. Strengthening epidemiological surveillance through the expansion of systems for monitoring the social determinants of health, implementing modern analytical tools, and training staff in interdisciplinary data analysis.
4. Implementation of health policies that take social determinants of health into account, based on the principles of health equity, structural prevention, and systemic adaptability, in line with the “health in all policies” approach.

Realization of this mandate is essential for building resilient, equitable, and sustainable healthcare systems capable of effectively responding to the complex challenges of contemporary polycrises. Social epidemiology, as a scientific tool integrating knowledge on inequalities, social mechanisms, and population health dynamics, should be treated not as optional support but as the foundation of health policy in the 21st century. The guiding role of social epidemiology in shaping resilient and equitable healthcare systems has also been emphasized in studies analyzing the impact of pandemic-related restrictions on patients and their families [99]. Therefore, to ensure that the response to polycrises is effective and fair, it is necessary to systematically incorporate social epidemiology into the analysis of each area. Such an approach makes it possible to fully understand the dynamics of health inequalities and to design policies that not only respond to crises, but also strengthen social and systemic resilience.

## 7. Summary

The coexistence and mutual reinforcement of crises—health, climate, economic, and geopolitical—create an amplification effect that significantly burdens healthcare systems. These crises not only accumulate over time but also interact with each other in a systemic way, leading to deepening health inequalities, overloading medical infrastructure, destabilization of public health management mechanisms, and limited access to services for the most vulnerable social groups. As a result, healthcare systems are becoming increasingly less capable of responding to complex and dynamic threats, which require not only adaptive solutions but also a structural transformation based on scientific knowledge.

To effectively mitigate the effects of polycrisis, coordinated international action is imperative. Collaboration among countries, international organizations, and civil society is essential to provide the resources, knowledge, and support necessary to fortify healthcare systems.

Incorporating social epidemiology, which investigates the influence of social factors on health, is crucial for understanding and addressing health inequalities. This approach enables the identification of the most vulnerable groups and the development of intervention strategies that consider the specific needs of diverse communities.

## Data Availability

Not applicable.

## References

[1] McNamara C., Bambra C. (2025). The Global Polycrisis and Health Inequalities. Int. J. Soc. Determ. Health Health Serv..

[2] Lawrence M., Homer-Dixon T., Janzwood S., Rockstöm J., Renn O., Donges J.F. (2024). Global polycrisis: The causal mechanisms of crisis entanglement. Glob. Sustain..

[3] Berkman L.F., Kawachi I., Glymour M.M. (2014). Social Epidemiology.

[4] Rutter P.D., Mytton O.T., Mak M., Donaldson L.J. (2012). Socio-economic disparities in mortality due to pandemic influenza in England. Int. J. Public Health.

[5] Lowcock E.C., Rosella L.C., Foisy J., McGeer A., Crowcroft N. (2012). The social determinants of health and pandemic H1N1 2009 influenza severity. Am. J. Public Health.

[6] Biggerstaff M., Jhung M.A., Reed C., Garg S., Balluz L., Fry A.M., Finelli L. (2014). Impact of medical and behavioural factors on influenza-like illness, healthcare-seeking, and antiviral treatment during the 2009 H1N1 pandemic. Epidemiol. Infect..

[7] Fallah M.P., Skrip L.A., Gertler A., Yamin D., Galvani A.P. (2015). Quantifying poverty as a driver of Ebola transmission. PLoS Negl. Trop. Dis..

[8] Amri M.M., Drummond D. (2020). Punctuating the equilibrium: An application of policy theory to COVID-19. Policy Des. Pract..

[9] Ledesma J.R., Chrysanthopoulou S.A., Lurie M.N., Nuzzo J.B., Papanicolas I. (2024). Health system resilience during the COVID-19 pandemic: A comparative analysis of disruptions in care from 32 countries. Health Serv. Res..

[10] Menendez C., Gonzalez R., Donnay F., Leke R.G.F. (2020). Avoiding the indirect effects of COVID-19 on maternal and child health. Lancet Glob. Health.

[11] Makoni M. (2020). Africa prepares for coronavirus. Lancet.

[12] Haileamlak A. (2021). The impact of COVID-19 on health and health systems. Ethiop. J. Health Sci..

[13] (2021). Department of Health and Social Care; Office for National Statistics; Government Actuary’s Department; Home Office. Direct and Indirect Impact of COVID-19 on Excess Deaths and Morbidity. https://assets.publishing.service.gov.uk/government/uploads/system/uploads/attachment_data/file/907616/s0650-direct-indirect-impacts-covid-19-excess-deaths-morbidity-sage-48.pdf.

[14] Ashbury F.D. (2021). COVID-19 and supportive cancer care: Key issues and opportunities. Curr. Opin. Oncol..

[15] Bardet A., Fraslin A.M., Marghadi J., Borget I., Faron M., Honoré C., Delaloge S., Albiges L., Planchard D., Ducreux M. (2021). Impact of COVID-19 on healthcare organizations and cancer outcomes. Eur. J. Cancer.

[16] Tille F., Van Ginneken E., Winkelmann J., Hernandez-Quevedo C., Falkenbach M., Sagan A., Karanikolos M., Cylus J. (2023). Perspective: Lessons from COVID-19 in countries in the European region in light of findings from the health system response monitor. Front. Public Health.

[17] Rajan S., McKee M., Hernández-Quevedo C., Karanikolos M., Richardson E., Webb E., Cylus J. (2022). What have European countries done to prevent the spread of COVID-19? Lessons from the COVID-19 Health system response monitor. Health Policy.

[18] Waitzberg R., Hernández-Quevedo C., Bernal-Delgado E., Estupiñán-Romero F., Angulo-Pueyo I., Theodorou M., Kantaris M., Charalambous C., Gabriel E., Economou C. (2022). Early health system responses to the COVID-19 pandemic in Mediterranean countries: A tale of success and challenges. Health Policy.

[19] Winkelmann J., Webb E., Williams G.A., Hernández-Quevedo C., Maier C.B., Panteli D. (2022). European countries’ responses in ensuring sufficient physical infrastructure and workforce capacity during the first COVID-19 wave. Health Policy.

[20] Falkenbach M., Willison C. (2022). Resources or trust: What matters more in the vaccination strategies of high-income liberal democracies?. Health Policy Technol..

[21] Antonini M., Eid M.A., Falkenbach M., Rosenbluth S.T., Prieto P.A., Brammli-Greenberg S., McMeekin P., Paolucci F. (2022). An analysis of the COVID-19 vaccination campaigns in France, Israel, Italy and Spain and their impact on health and economic outcomes. Health Policy Technol..

[22] Mosadeghrad A.M., Afshari M., Isfahani P., Ezzati F., Abbasi M., Farahani S.A., Zahmatkesh M., Eslambolchi L. (2024). Strategies to strengthen the resilience of primary healthcare in the COVID-19 pandemic: A scoping review. BMC Health Serv. Res..

[23] Ezzati F., Mosadeghrad A.M., Jaafaripooyan E. (2023). Resiliency of the Iranian healthcare facilities against the Covid-19 pandemic: Challenges and solutions. BMC Health Serv. Res..

[24] Sagan A., Thomas S., McKee M., Karanikolos M., Azzopardi-Muscat N., de la Mata I., Figueras J. (2020). COVID-19 and Health Systems Resilience: Lessons Going Forwards. Eurohealth.

[25] World Health Organization (2021). Building Health Systems Resilience for Universal Health Coverage and Health Security During the COVID-19 Pandemic and Beyond: WHO Position Paper.

[26] Sachs J.D., Lafortune G., Fuller G., Iablonovski G. (2025). Financing Sustainable Development to 2030 and the Mid-Century.

[27] Uppsala University. UCDP: Record Number of Armed Conflicts in the World. 2024. https://www.uu.se/en/press/press-releases/2024/2024-06-03-ucdp-record-number-of-armed-conflicts-in-the-world.

[28] World Population Review. Countries Currently at War. 2025. https://worldpopulationreview.com/country-rankings/countries-currently-at-war.

[29] Jayasinghe S. (2024). The 12 dimensions of health impacts of war (the 12-D framework): A novel framework to conceptualise impacts of war on social and environmental determinants of health and public health. BMJ Glob. Health.

[30] Ramadan M., Tappis H., Uribe M.V., Brieger W. (2021). Access to primary healthcare Services in Conflict-Affected Fragile States: A subnational descriptive analysis of educational and wealth disparities in Cameroon, Democratic Republic of Congo, Mali, and Nigeria. Int. J. Equity Health.

[31] Amberg F., Blanchet K., Singh N.S., Ridde V., Bonnet E., Yaméogo P., Sie A., Seynou M., Lohmann J., De Allegri M. (2025). Examining the effect of nearby armed conflict on access to maternal and child health services in Burkina Faso’s primary healthcare facilities. BMJ Glob. Health.

[32] Ridde V., Yaméogo P. (2018). How Burkina Faso used evidence in deciding to launch its policy of free healthcare for children under five and women in 2016. Palgrave Commun..

[33] Bicaba F., Browne L., Kadio K., Bila A., Bicaba A., Druetz T. (2020). National user fee abolition and health insurance scheme in Burkina Faso: How can they be integrated on the road to universal health coverage without increasing health inequities?. J. Glob. Health.

[34] Price J.I., Bohara A.K. (2013). Maternal health care amid political unrest: The effect of armed conflict on antenatal care utilization in Nepal. Health Policy Plan..

[35] Chukwuma A., Ekhator-Mobayode U.E. (2019). Armed conflict and maternal health care utilization: Evidence from the Boko Haram Insurgency in Nigeria. Soc. Sci. Med..

[36] Namasivayam A., Arcos González P., Castro Delgado R., Chi P.C. (2017). The effect of armed conflict on the utilization of maternal health services in Uganda: A population-based study. PLoS Curr. Disasters.

[37] Yaya S., Uthman O.A., Bishwajit G., Ekholuenetale M. (2019). Maternal health care service utilization in post-war Liberia: Analysis of nationally representative cross-sectional household surveys. BMC Public Health.

[38] Brentlinger P.E., Sánchez-Pérez H.J., Arana Cedeño M., Morales L.G.V., Hernán M.A., Micek M.A., Ford D. (2005). Pregnancy outcomes, site of delivery, and community schisms in regions affected by the armed conflict in Chiapas, Mexico. Soc. Sci. Med..

[39] Ekzayez A., Alhaj Ahmad Y., Alhaleb H., Checchi F. (2021). The impact of armed conflict on utilisation of health services in north-west Syria: An observational study. Confl. Health.

[40] Orcutt M., Rayes D., Tarakji A., Katoub M., Spiegel P., Rubenstein L., Jabbour S., Alkhalil M., Alabbas M., Abbara A. (2019). International failure in northwest Syria: Humanitarian health catastrophe demands action. Lancet.

[41] Alameddine M., Fouad F.M., Diaconu K., Jamal Z., Lough G., Witter S., Ager A. (2019). Resilience capacities of health systems: Accommodating the needs of Palestinian refugees from Syria. Soc. Sci. Med..

[42] Rayes D., Orcutt M., Abbara A., Maziak W. (2018). Systematic destruction of healthcare in Eastern Ghouta, Syria. BMJ.

[43] Spagat M. (2018). Attacks on medical workers in Syria: Implications for conflict research. PLoS Med..

[44] Fouad F.M., Sparrow A., Tarakji A., Alameddine M., El-Jardali F., Coutts A.P., El Arnaout N., Karroum L.B., Jawad M., Roborgh S. (2017). Health workers and the weaponisation of health care in Syria: A preliminary inquiry for The Lancet–American University of Beirut Commission on Syria. Lancet.

[45] Horton R. (2023). Offline: The case for a global health board. Lancet.

[46] Druce P., Bogatyreva E., Siem F.F., Gates S., Kaade H., Sundby J., Rostrup M., Andersen C., Rustad S.C.A., Tchie A. (2019). Approaches to protect and maintain healthcare services in armed conflict: Meeting SDGs 3 and 16. Confl. Health.

[47] International Committee of the Red Cross. The Geneva Conventions and Their Commentaries. 1949. https://www.icrc.org/en/war-and-law/treaties-customary-law/geneva-conventions.

[48] United Nations Security Council. Security Council Adopts Resolution 2286 (2016), Strongly Condemning Attacks Against Medical Facilities, Personnel in Conflict Situations. 2016. https://www.un.org/press/en/2016/sc12347.doc.htm.

[49] Brennan R. (2024). The international community is failing to protect healthcare in armed conflict. BMJ.

[50] World Health Organization WHO Strategic and Technical Advisory Group for Antimicrobial Resistance (STAG-AMR). https://extranet.who.int/ssa/LeftMenu/Index.aspx.

[51] Gavi, the Vaccine Alliance (2023). Attacks on Health Care During War Are Becoming More Common, Creating Devastating Ripple Effects. https://www.gavi.org/vaccineswork/attacks-health-care-during-war-are-becoming-more-common-creating-devastating-ripple.

[52] World Health Organization (2025). People in Gaza Starving, Sick and Dying as Aid Blockade Continues. https://www.who.int/news/item/12-05-2025-people-in-gaza-starving--sick-and-dying-as-aid-blockade-continues.

[53] (2025). Integrated Food Security Phase Classification. IPC Gaza Strip Acute Food Insecurity Analysis, April–September 2025. https://www.ipcinfo.org/fileadmin/user_upload/ipcinfo/docs/IPC_Gaza_Strip_Acute_Food_Insecurity_Apr_Sep2025_Special_Report.pdf.

[54] Asmad I. (2025). The Devastating Effects of Gaza War on Healthcare. East. Mediterr. Health J..

[55] Ghobarah H.A., Huth P., Russett B. (2004). The Post-War Public Health Effects of Civil Conflict. Bull. World Health Organ..

[56] Kruk M.E., Myers M., Varpilah S.T., Dahn B.T. (2015). What Is a Resilient Health System? Lessons from Ebola. Lancet.

[57] Blanchet K., Sistenich V., Ramesh A., Frison S., Warren E., Smith J., Hossain M., Knight A., Lewis C., Post N. (2015). An Evidence Review of Research on Health Interventions in Humanitarian Crises.

[58] World Health Organization (2023). Climate Change and Health. https://www.who.int/news-room/fact-sheets/detail/climate-change-and-health.

[59] Al-Marwani S. (2023). Climate change impact on the healthcare provided to patients. Bull. Natl. Res. Cent..

[60] Southerland V.A., Brauer M., Mohegh A., Hammer M.S., Van Donkelaar A., Martin R.V., Apte J.S., Anenberg S.C. (2022). Global urban temporal trends in fine particulate matter (PM2.5) and attributable health burdens: Estimates from global datasets. Lancet Planet Health.

[61] Monteiro A., Carvalho V., Oliveira T., Sousa C. (2013). Excess mortality and morbidity during the July 2006 heat wave in Porto, Portugal. Int. J. Biometeorol..

[62] Correa-Rotter R., Wesseling C., Johnson R.J. (2014). CKD of unknown origin in Central America: The case for a Mesoamerican nephropathy. Am. J. Kidney Dis..

[63] Conrad K. (2023). The Era of Climate Change Medicine: Challenges to Healthcare Systems. Ochsner J..

[64] Pörtner H.-O., Roberts D.C., Tignor M., Poloczanska E.S., Mintenbeck K., Alegría A., Craig M., Langsdorf S., Löschke S., Möller V., Intergovernmental Panel on Climate Change (IPCC) (2022). Climate Change 2022: Impacts, Adaptation and Vulnerability.

[65] Gerwig K. (2022). Climate change and healthcare: A complicated relationship. Front. Health Serv. Manag..

[66] Salas R.N., Friend T.H., Bernstein A., Jha A.K. (2020). Adding a climate lens to health policy in the United States. Health Aff..

[67] Fouillet A., Rey G., Laurent F., Pavillon G., Bellec S., Guihenneuc-Jouyaux C., Clavel J., Jougla E., Hémon D. (2006). Excess mortality related to the August 2003 heat wave in France. Int. Arch. Occup. Environ. Health.

[68] Nogueira L.M., Sahar L., Efstathiou J.A., Jemal A., Yabroff K.R. (2019). Association between declared hurricane disasters and survival of patients with lung cancer undergoing radiation treatment. J. Am. Med. Assoc..

[69] MacFadden D.R., McGough S.F., Fisman D., Santillana M., Brownstein J.S. (2018). Antibiotic resistance increases with local temperature. Nat. Clim. Change.

[70] Layton J.B., Li W., Yuan J., Gilman J.P., Horton D.B., Setoguchi S. (2020). Heatwaves, medications, and heat-related hospitalization in older Medicare beneficiaries with chronic conditions. PLoS ONE.

[71] Or Z., Seppänen A.-V. (2024). The Role of the Health Sector in Tackling Climate Change: A Narrative Review. Health Policy.

[72] Reed T. Kaiser Permanente’s Health System Reaches Carbon-Neutral Status. Fierce Healthcare, Published 15 September 2020. https://www.fiercehealthcare.com/hospitals/kaiser-permanente-s-heath-system-reaches-carbon-neutral-status.

[73] Jennings N., Rao M. (2020). Towards a carbon neutral NHS. BMJ.

[74] Recio R.S., Alonso Pérez De Ágreda J.P., Rabanaque M.J., Aguilar Palacio I. (2022). Understanding the Effect of Economic Recession on Healthcare Services: A Systematic Review. Iran J. Public Health.

[75] Katikireddi S.V., Niedzwiedz C.L., Popham F. (2012). Trends in population mental health before and after the 2008 recession: A repeat cross-sectional analysis of the 1991–2010 Health Surveys of England. BMJ Open.

[76] Economou M., Madianos M., Theleritis C., Peppou L., Stefanis C. (2011). Increased suicidality amid economic crisis in Greece. Lancet.

[77] Houdmont J., Kerr R., Addley K. (2012). Psychosocial factors and economic recession: The Stormont Study. Occup. Med..

[78] Barr B., Taylor-Robinson D., Scott-Samuel A., McKee M., Stuckler D. (2012). Suicides associated with the 2008—10 economic recession in England: Time Trend analysis. BMJ.

[79] Bambra C., Garthwaite K., Copeland A., Barr B., Smith K.E., Hill S., Bambra C. (2016). All in it together? Health inequalities, welfare austerity and the ‘great recession’. Health Inequalities: Critical Perspectives.

[80] Sánchez-Recio R., Alonso J.P., Rabanaque M.J., Aguilar-Palacio I. (2021). Further information about the research “Understanding the effect of economic recession on healthcare services: A systematic review”. Figshare.

[81] Milionis C. (2013). Provision of healthcare in the context of financial crisis: Approaches to the Greek health system and international implications. Nurs. Philos..

[82] da Costa F.A., Teixeira I., Duarte-Ramos F., Proença L., Pedro A.R., Furtado C., da Silva J.A., Cabrita J. (2017). Effects of economic recession on elderly patients’ perceptions of access to health care and medicines in Portugal. Int. J. Clin. Pharm..

[83] Karanikolos M., Mladovsky P., Cylus J., Thomson S., Basu S., Stuckler D., Mackenbach J.P., McKee M. (2013). Financial crisis, austerity, and health in Europe. Lancet.

[84] Lopez-Valcarcel B.G., Barber P. (2017). Economic crisis, austerity policies, health and fairness: Lessons learned in Spain. Appl. Health Econ. Health Policy.

[85] Kondilis E., Giannakopoulos S., Gavana M., Ierodiakonou I., Waitzkin H., Benos A. (2013). Economic crisis, restrictive policies, and the population’s health and health care: The Greek case. Am. J. Public Health.

[86] Israel S. (2016). How social policies can improve financial accessibility of healthcare: A multilevel analysis of unmet medical need in European countries. Int. J. Equity Health.

[87] Glassman A., Madan Keller J., Smitham E. (2023). The Future of Global Health Spending Amidst Multiple Crises.

[88] Revill P., Glassman A. (2019). Understanding the Opportunity Cost, Seizing the Opportunity: Report of the Working Group on Incorporating Economics and Modelling in Global Health Goals and Guidelines.

[89] Russo G., Pires C.A., Perelman J., Gonçalves L., Barros P.P. (2017). Exploring public sector physicians’ resilience, reactions and coping strategies in times of economic crisis: Findings from a survey in Portugal’s capital city area. BMC Health Serv. Res..

[90] Cylus J., Pearson M. (2015). The crisis and its implications for household financial security, government resources and health expenditure. Economic Crisis, Health Systems and Health in Europe: Impact and Implications for Policy.

[91] Sriram V., Yilmaz V., Kaur S., Andres C., Cheng M., Meessen B. (2024). The Role of Private Healthcare Sector Actors in Health Service Delivery and Financing Policy Processes in Low- and Middle-Income Countries: A Scoping Review. BMJ Glob. Health.

[92] Burström B. (2015). The attack on universal health coverage in Europe: Different effects in different parts of Europe. Eur. J. Public Health.

[93] Foroughi Z., Ebrahimi P., Yazdani S., Aryankhesal A., Heydari M., Maleki M. (2025). Analysis for health system resilience against the economic crisis: A best-fit framework synthesis. Health Res. Policy Syst..

[94] Phelan J.C., Link B.G., Tehranifar P. (2010). Social Conditions as Fundamental Causes of Health Inequalities: Theory, Evidence, and Policy Implications. J. Health Soc. Behav..

[95] Rudd K.E., Mair C.F., Angus D.C. (2022). Applying Syndemic Theory to Acute Illness. J. Am. Med. Assoc..

[96] Steinbeis F., Gotham D., von Philipsborn P., Stratil J.M. (2019). Quantifying Changes in Global Health Inequality: The Gini and Slope Inequality Indices Applied to the Global Burden of Disease Data, 1990–2017. BMJ Glob. Health.

[97] El-Sayed A.M., Scarborough P., Seemann L., Galea S. (2012). Social Network Analysis and Agent-Based Modeling in Social Epidemiology. Epidemiol. Perspect. Innov..

[98] Minkler M., Garcia A.P., Rubin V., Wallerstein N. (2012). Community-Based Participatory Research: A Strategy for Building Healthy Communities and Promoting Health through Policy Change.

[99] Jaroń K., Grajek M., Kobza J. (2025). Impact of COVID-19 Visitation Restrictions on Hospitalized Patients and Their Families—A Dual Perspective. Ann. Acad. Med. Siles..

